# Systemic Risk Factors for Adult Spinal Deformity (ASD): A Retrospective Analysis of 48 Patients

**DOI:** 10.7759/cureus.25214

**Published:** 2022-05-22

**Authors:** Nicholas Dietz, Peter Hollis, Enzo Fortuny, Basil Gruter, Justin Virojanapa, Brian Williams, Alexander Spiessberger

**Affiliations:** 1 Neurosurgery, University of Louisville School of Medicine, Louisville, USA; 2 Neurosurgery, North Shore University Hospital, Manhasset, USA; 3 Neurosurgery, University of Switzerland, Zurich, CHE

**Keywords:** spinopelvic parameters, pelvic incidence, sagittal vertical axis, risk factors, adult spinal deformity

## Abstract

Introduction: Adult spinal deformity (ASD) results in significant patient morbidity and burden to quality of life. The degree to which systemic risk factors and comorbidities that contribute to ASD affect specific spinopelvic parameters is not well-documented. We determine the extent to which preoperative risk factors may contribute to spinopelvic parameters associated with ASD.

Methods: Retrospective single-center study of 48 patients with ASD. Analysis of variance (ANOVA) linear regression analysis was performed to evaluate correlation between systemic comorbidities (obesity, arterial hypertension (HTN), hyperlipidemia (HLD), cardiomyopathy, diabetes mellitus (DM), chronic obstructive pulmonary disease (COPD), and asthma) and the following radiographic parameters: pelvic incidence (PI), lumbar lordosis (LL), C7 sagittal vertical axis (C7SVA), and the T10-L2 sagittal cobb angle.

Results: A total of 48 patients were included with mean C7SVA of 79.6 mm (SD: 63, range: 43-254), mean LL of 32.9° (SD: 15.9, range: -14 to 78), T10-L2 sagittal cobb angle of 3° (SD: 12.7, range: -24 to 30), and PI was 49° (SD: 10.7, range: 21 to 77). Only DM correlated with sagittal imbalance with high C7SVA and PI-LL mismatch. The beta coefficient for DM and preoperative C7SVA was 0.49, t=3.16, p=0.003, preoperative PI-LL mismatch standardized beta coefficient was -0.4, t=-2.38, p=0.022, and preoperative T10-L2 sagittal cobb standard beta coefficient was -0.07, t=-0.46, p=0.645. No significant correlations were found for asthma, COPD, HTN, HLD, or cardiomyopathy.

Conclusions: Diagnosis of DM was found to correlate with pathologic C7SVA and significant PI-LL mismatch associated with ASD. HTN, HLD, cardiomyopathy, obesity, and pulmonary disease did not correlate with radiographic findings of sagittal imbalance.

## Introduction

Adult spinal deformity (ASD) represents a significant burden to quality of life, contributing to pain and disability as well as economic strain on the individual and healthcare system [1-3]. Prevalence of ASD in older adults may be as high as 68%, and is expected to increase given the aging population [1,4,5]. Several etiologies contribute to ASD such as degenerative spine disease, progression of adolescent idiopathic scoliosis (AIS), previous spine surgery, infection, vertebral fracture, and malignancy [1,6,7].

The present study focuses on the most common form of ASD, which is characterized by thoracolumbar sagittal imbalance with or without de novo scoliosis. In this type of deformity, multi-level disc degeneration leads to loss of disc height, which in turn leads to loss of segmental lordosis and potentially coronal deformity from asymmetric disc degeneration [1,2]. In addition, degenerative changes of the disc frequently lead to degeneration of the facet joints, causing foraminal and spinal stenosis and spondylolisthesis [8,9]. Known risk factors for disc degeneration, the core process driving the development of adult deformity, are genetic factors, elevated body mass index (BMI), older age, occupational, and overused axial load bearing [10-13]. Additionally, cardiovascular disease and atherosclerosis risk factors such as high low-density lipoproteins (LDLs) profiles may underlie disc degeneration [12,14,15]. Insufficient blood supply to the disc through microangiopathy from smoking or systemic vascular diseases such as diabetes mellitus (DM) may also be contributory [16,17].

This study aims to investigate possible systemic factors contributing to the development of ASD and which alignment parameters may be most affected. As healthcare management progresses toward multi-modal and integrated platforms, knowledge of modifiable and non-modifiable risk factors of this increasingly common disease in older adults may inform best practices for prevention and treatment.

## Materials and methods

Approval for this study was obtained by the authors’ institutional review board (IRB 20-0444). Patients with ASD undergoing surgical treatment between January 1, 2015 and January 31, 2020 were included from an institutional surgical database if they met the following criteria: 1) etiology of deformity other than focal pathologies (e.g. fractures, tumors); 2) complete radiographic evaluation consisting of standing long film x rays; and 3) age greater than 18 years. The following demographic information was extracted for the patients: age at index surgery, sex, BMI, comorbidities: arterial hypertension (HTN), hyperlipidemia (HLD), cardiomyopathy, DM type, COPD or asthma. Standing x-rays of the thoracolumbar spine from C7 to the acetabula were reviewed and the following measurements extracted for each patient: pelvic incidence (PI), lumbar lordosis (LL), C7 sagittal vertical axis (C7SVA), and the T10-L2 sagittal cobb angle [18].

Statistical analysis was performed using PSPP (GNU PSPP (2015), Free Software Foundation Boston, MA). Analysis of variance (ANOVA) linear regression analysis was performed with the following dependent variables: BMI, HTN, HLD, cardiomyopathy, DM, COPD/asthma. Radiographic parameters investigated were C7SVA, PI-LL mismatch, and T10-L2 sagittal cobb angle.

## Results

A total of 48 patients were included in the present analysis, 63% of them being female and a mean age of 68.7 years (SD: 8.6, range: 39.9 to 84.7) (Table 1). The cohort investigated had the following comorbidities of relevance for this study: HTN in 31 patients (65%), HLD in 10 patients (21%), cardiomyopathy in eight patients (17%), DM in 19 patients (31%), and asthma or COPD in nine patients (19%). The mean BMI was 30.8 (SD: 6.3, range: 21 to 46).

**Table 1:** Patient demographics SD: Standard deviation; HTN: Hypertension; HLD: Hyperlipidemia; DM: Diabetes mellitus

Number of patients	48
Age (SD, range)	68.7, (8.6, 39.9–84.7)
Sex (female)	63%
BMI (SD, range)	30.8, (6.3, 21–46)
HTN	65%
HLD	21%
Cardiomyopathy	17%
DM	31%
Asthma, COPD	19%

Radiographic parameters of the study cohort are shown in Table 2. Mean C7SVA was 79.6 mm (SD: 63, range: -43 to 254), mean LL was 32.9° (SD: 15.9, range: -14 to 78), T10-L2 sagittal cobb angle was 3° (SD: 12.7, range: -24 to 30), and PI was 49° (SD: 10.7, range: 21 to 77).

**Table 2:** Radiographic measures C7SVA: C7 sagittal vertical axis; SD: Standard deviation; LL: Lumbar lordosis; PI: Pelvic incidence

Measure	Value
C7SVA (mm) (SD, range)	79.6, (63, -43–254)
LL (degree) (SD, range)	32.9, (15.9, -14–78)
T10-L2 sagittal cobb angle (degree) (SD, range)	3, (12.7, -24–30)
PI (degree) (SD, range)	49, (10.7, 21–77)

For DM, the beta coefficient for preoperative C7SVA was 0.49, t=3.16, p=0.003; preoperative PI-LL mismatch standardized beta coefficient of -0.4, t=-2.38, p=0.022; and preoperative T10-L2 sagittal cobb standard beta coefficient of -0.07, t=-0.46, p=0.645 (see Table 3). In asthma and COPD, preoperative C7SVA was 0.00, t= 1.72, p=0.093; preoperative PI-LL mismatch standardized beta coefficient of 0.27, t=0.05, p=0.959; and preoperative T10-L2 sagittal cobb standard beta coefficient of -0.04, t=-0.76, p=0.452. For HTN, preoperative C7SVA was 0.11, t=0.67, p=0.507; preoperative PI-LL mismatch was -0.07, t=-0.41, p=0.683; and preoperative T10-L2 sagittal cobb was -0.25, t=-1.47, p=0.150. In patients with HLD, preoperative C7SVA was -0.27, t=-1.66, p=0.104; preoperative PI-LL mismatch was -0.22, t=-1.24, p=0.222; and preoperative T10-L2 sagittal cobb was 0.22, t=-1.33, p=0.190. For cardiomyopathy, preoperative C7SVA was 0.14, t= 0.84, p=0.405; preoperative PI-LL mismatch was 0.18, t=1.01, p=0.321; and preoperative T10-L2 sagittal cobb was -0.11, t=-0.68, p=0.503.

**Table 3:** Correlation of risk factors for deformity and spinopelvic parameter measures BMI: Body mass index; C7SVA: C7 sagittal vertical axis; PI: Pelvic incidence; LL: Lumbar lordosis; DM: Diabetes mellitus; COPD: Chronic obstructive pulmonary disease; HTN: Hypertension; HLD: Hyperlipidemia

BMI	Unstandardized coefficients		Standardized coefficient		
	B	Standard error	beta	t	significance
preop C7SVA	0	0.02	0	-0.01	0.99
preop PI-LL mismatch	0	0.06	0.27	1.5	0.141
preop T10-L2 sagittal cobb	-0.02	0.09	-0.04	-0.25	0.806
DM (Type II)	Unstandardized coefficients		Standardized coefficient		
	B	Standard error	beta	t	significance
preop C7SVA	0	0	0.49	3.16	0.003
preop PI-LL mismatch	-0.01	0	-0.4	-2.38	0.022
preop T10-L2 sagittal cobb	0	0.01	0.07	0.46	0.645
Asthma, COPD	Unstandardized coefficients		Standardized coefficient		
	B	Standard error	beta	t	significance
preop C7SVA	0	0	0	1.72	0.093
preop PI-LL mismatch	0	0	0.27	0.05	0.959
preop T10-L2 sagittal cobb	0	0.01	-0.04	-0.76	0.452
HTN	Unstandardized coefficients		Standardized coefficient		
	B	Standard error	beta	t	significance
preop C7SVA	0	0	0.11	0.67	0.507
preop PI-LL mismatch	0	0	-0.07	-0.41	0.683
preop T10-L2 sagittal cobb	-0.01	0.01	-0.25	-1.47	0.15
HLD	Unstandardized coefficients		Standardized coefficient		
	B	Standard error	beta	t	significance
preop C7SVA	0	0	-0.27	-1.66	0.104
preop PI-LL mismatch	0	0	-0.22	-1.24	0.222
preop T10-L2 sagittal cobb	0.01	0.01	0.22	1.33	0.19
Cardiomyopathy	Unstandardized coefficients		Standardized coefficient		
	B	Standard error	beta	t	significance
preop C7SVA	0	0	0.14	0.84	0.405
preop PI-LL mismatch	0	0	0.18	1.01	0.321
preop T10-L2 sagittal cobb	0	0.01	-0.11	-0.68	0.503

## Discussion

In this retrospective study, DM has been the only systemic comorbidity, which correlated with radiographic sagittal imbalance, the hallmark of ASD. A variety of spinopelvic parameters are used to assess ASD, however C7SVA, PI-LL mismatch, and pelvic tilt (PT) have been shown to relate strongly to patient reported outcomes and quality of life [19]. Risk factors associated with the development of structural spine element degeneration and ASD include low bone mineral density and high BMI [3,9,20]. Disc and facet degeneration, leading underlying causes of sagittal and coronal deformity in primary or de novo ASD, contribute to progressive and insidious imbalance in multiple planes of the spinal column [20]. Recent evidence supports the deleterious effects of elevated serum lipid levels and inflammation to disc degeneration [12,14]. Compromised vascular supply from increased burden of atherosclerosis is believed to be involved in the pathophysioliogy [21,22]. As such, other systemic conditions that may serve as burdens to vascular supply, such as HTN, DM, and cardiomyopathy, may also increase disc degeneration and ASD incidence.

The present study identified key comorbid risk factors related to sub-measures of spinopelvic parameters of ASD. Among the comorbidities studied, including obesity, DM, asthma or COPD, HTN, HLD, and cardiomyopathy, only DM was found to correlate with sagittal imbalance in the forms of high C7SVA and PI-LL mismatch. In a prospective multicenter study, Schwab and colleagues identify PI-LL mismatch greater than 10 degrees [23] relates to increased likelihood of disability and poor quality of life in patients with ASD [24]. Additionally, Ghandari and colleagues show that PI-LL mismatch is the most important parameter in relation postoperative outcome as its preoperative to postoperative change value was most correlated with improvement in Oswestry Disability Index [25]. Higher C7SVA is also known to increase compensatory workload and contribute to pain associated with ASD [23,26]. Indeed, restoration of SVA is among the primary goals of surgical correction of ASD to less than 47 mm [24]. These particular parameters may therefore be particularly important in the evaluation for severity and surgical amenability of ASD.

Arteriosclerosis is known to be implicated in the development of degenerative disc disease [12,14]. Previous studies demonstrate a correlation between arteriosclerosis of the lumbar arteries and radiographic disc degeneration as well as lower back pain [14,21]. Given that HTN and HLD are risk factors for the development of arteriosclerosis [27-29], we also investigated the possible correlation between these two factors and sagittal imbalance. Duration and degree of HTN and elevated LDLs may influence degree of arteriosclerosis and atherosclerosis. It is possible that HTN and HLD were not correlated with sagittal imbalance in our sample because of an insufficient atherosclerotic or arteriosclerotic burden that would lead to degenerative disc disease.

Studies suggest that DM accelerates the development of degenerative disc disease [30]. The present study further demonstrates that the duration of diabetes had an influence on the grade of disc degeneration [30]. One theory regarding the influence of diabetes on development of multisegmental degenerative disc changes is premature and excessive degeneration of nucleus pulpous from advanced glycation end products [31-34]. As a systemic metabolic disease, diabetes may also have a disproportionate effect on vascular supply and arteriosclerosis. A micorangiopathy may be associated with DM like that of smoking that portends higher rates of multisegmental disc degeneration [35,36].

Previous studies have demonstrated a correlation between obesity and back pain [37-39]. However, a correlation between radiographically advanced disc degeneration, a prerequisite in the development of adult deformity, has not been made. Our study failed to show a correlation between radiographic sagittal imbalance, in which identifiable multisegmental disc degeneration is a prerequisite for development of ASD. Radiographs may not always provide sufficient evidence of significant disc injury or spondylosis, and even MRI may have limited value in diagnosis of early degenerative disc disease [40,41].

A strength of the current study is its design using a single-center retrospective analysis to standardize methods of diagnosis, documentation, imaging, and clinical care. Future studies may also incorporate MRI analysis to evaluate degree of degenerative disc disease, considering higher sensitivity than CT and plain radiographs [40]. Our study also benefits from previous research on ASD to isolate particular spinopelvic parameters known to be indicative of disability and clinical function for this patient population. One limitation may be a lack of longitudinal analysis to determine postoperative changes as they may relate to preoperative measures and comorbidities. Future studies may also examine the influence of risk factors such as DM on patients postoperatively, affecting outcomes, proximal junctional kyphosis, or need for reoperation. Specifically, analysis of SVA abnormality and the extent and duration of controlled versus uncontrolled diabetes using latest HbA1c values, for example, may offer more insight into preoperative clinical management of DM in ASD. Further, Additionally, bone mineral density to assess osteoporosis, another factor known to be associated with ASD, would have been a good variable to include as a comorbid risk factor, but was not retrievable in the current analysis.

## Conclusions

Diagnosis of DM was found to correlate with pathologic C7SVA and significant PI-LL mismatch associated with ASD. HTN, HLD, cardiomyopathy, obesity, and pulmonary disease did not correlate with radiographic findings of sagittal imbalance. Given the clinical importance of these spinopelvic parameters, further investigation is warranted to evaluate imaging of disc degeneration severity, clinical history of DM, and postoperative performance for those patients who received correction for ASD.
